# Sequence analysis of capnography waveform abnormalities during nurse-administered procedural sedation and analgesia in the cardiac catheterization laboratory

**DOI:** 10.1038/s41598-019-46751-2

**Published:** 2019-07-15

**Authors:** Aaron Conway, Peter Collins, Kristina Chang, Sebastian Mafeld, Joanna Sutherland, James Fingleton

**Affiliations:** 1 Peter Munk Cardiac Centre, Toronto General Hospital, University Health Network, Toronto, Canada; 2 Lawrence S. Bloomberg Faculty of Nursing, University of Toronto, Toronto, Canada; 3 Institute of Health and Biomedical Innovation, School of Nursing, Queensland University of Technology, Brisbane, Australia; 4 Interventional Radiology, Toronto General Hospital, University Health Network, Toronto, Canada; 5 Department of Anaesthesia, Coffs Harbour Health Campus, Coffs Harbour, Australia; 6 Medical Research Institute of New Zealand, Wellington, New Zealand

**Keywords:** Health care, Therapeutics, Cardiology

## Abstract

Identifying common patterns in capnography waveform abnormalities and the factors that influence these patterns could yield insights to optimize responses to sedation-induced respiratory depression. Respiratory state sequences for 102 patients who had a procedure in a cardiac catheterisation laboratory with procedural sedation and analgesia were developed by classifying each second of procedures into a state of normal breathing or other capnography waveform abnormalities based on pre-specified cut-offs for respiratory rate and end-tidal CO_2_ concentration. Hierarchical clustering identified four common patterns in respiratory state sequences, which were characterized by a predominance of the state assigned normal breathing (n = 42; 41%), hypopneic hypoventilation (n = 38; 38%), apnea (n = 15; 15%) and bradypneic hypoventilation (n = 7; 7%). A multivariable distance matrix regression model including demographic and clinical variables explained 28% of the variation in inter-individual differences in respiratory state sequences. Obstructive sleep apnea (R^2^ = 2.4%; p = 0.02), smoking status (R^2^ = 2.8%; p = 0.01), Charlson comorbidity index score (R^2^ = 2.5%; p = 0.021), peak transcutaneous carbon dioxide concentration (R^2^ = 4.1%; p = 0.002) and receiving an intervention to support respiration (R^2^ = 5.6%; p = 0.001) were significant covariates but each explained only small amounts of the variation in respiratory state sequences. Oxygen desaturation (SpO_2_ < 90%) was rare (n = 3; 3%) and not associated with respiratory state sequence trajectories.

## Introduction

Cardiology procedures are often performed using nurse-administered procedural sedation and analgesia^1,2^. The medications used for sedation are central nervous system depressants. Known side effects include partial obstruction of the airways arising from relaxation of the surrounding musculature, reduced respiratory rate and depth of breathing as well as periods of apnea. These side effects can be collectively termed sedation-induced respiratory depression. Monitoring for respiratory depression is a particularly important aspect of the management of sedated patients and it is significantly more likely to be detected when capnography is used^3^. However, we recently identified in our systematic review that randomized controlled trials have produced conflicting results regarding whether capnography improves patient safety when used during sedation^4^. In this context, it is important to note that intervening at the onset of an episode of respiratory depression that would have resolved spontaneously in a short period of time may be counterproductive because frequent interruptions could result in inadequate sedation. Despite uncertainties regarding how capnography should be deployed in the moderate sedation context, it seems likely to become standard practice for monitoring ventilation of all sedated patients^5,6^. In this study we utilized an approach known as state sequence analysis to examine capnography waveform abnormalities throughout cardiology procedures performed with sedation and analgesia. Specifically, the study aimed to:

1. Identify common ways in which capnography waveform abnormalities manifest throughout procedures performed with sedation
2. Determine if particular patient characteristics were associated with capnography waveform abnormalities.
3. Determine if intra-procedural ventilation and oxygenation status were associated with capnography waveform abnormalities.
4. Determine if patient recovery outcomes were associated with capnography waveform abnormalities.
5. Determine if interventions used to support sedated patients’ breathing were associated with capnography waveform abnormalities.

## Methods

State sequence analysis combines descriptive and inferential statistics to examine inter-individual differences in sequence trajectories. There are various ways in which state sequences may differ, including the constituting states, the order in which states occur (termed sequencing), the time-point in the sequence that states occur (termed timing) and the time spent in the same state (termed duration)^7^. It is possible to examine inter-individual variation in these attributes of state sequences by creating a matrix of pairwise dissimilarities (termed a dissimilarity matrix), which quantifies differences in the timing, sequencing and duration of sequences.

To address the first aim of our study, which was to identify common ways in which capnography waveform abnormalities manifest throughout procedures performed with sedation, the dissimilarity matrix was used to classify sequences that were similar to each other into groups using clustering techniques. Regression analyses (termed multivariable distance matrix regression analysis or MDMRA) can also be used to examine associations between state sequences and other variables of interest. In the context of this research, we used MDMRA to assess whether respiratory state sequences were more similar in patients with particular demographic and clinical variables in order to address aims 2–5. The Uniting Care Health Human Research Ethics Committee (Record number: 1614) and the St Vincent’s Health and Aged Care Human Research Ethics Committee (Record number: 16/26) approved the study and it was undertaken in accordance with the National Statement on Ethical Conduct in Human Research^8^. All participants provided informed consent. The study was registered at the Australia and New Zealand Clinical Trials Registry (ACTRN12616001132437). It should be noted that we originally planned to recruit 180 participants and perform latent class analysis on the total durations of different capnography waveform abnormalities observed during procedures. A sample that is too small for latent class analysis may lead to choosing a solution where the number of classes (subgroups) does not adequately describe the data. For this reason, we considered that our final sample size was insufficient to use latent class analysis and instead used the state sequence approach described here instead. We considered that MDMRA would be an appropriate alternative considering it is an accepted and widely used statistical analysis approach for situations where there is a large number of predictors in comparison to the sample size^9^.

### Participants

Adult patients scheduled to undergo an elective procedure with moderate sedation (intravenous midazolam or fentanyl or midazolam and fentanyl) administered by nurses with direction from a cardiologist at two private hospitals in Brisbane, Australia were invited to participate unless they were cognitively impaired (due to inability to provide informed consent) or unable to understand and speak English (and an interpreter was unavailable).

### Data collection

As is recommended for procedural sedation research^10^, a combination of interactive and physiological monitoring data was collected during the procedure by one of the researchers (performed for all procedures by AC). The researcher was present in the procedure room because direct observation of the participant as well as of any interventions applied by sedation providers was required. All routine clinical monitoring was applied by the nursing staff (i.e. electrocardiography, non-invasive blood pressure and pulse oximetry). Data concerning demographics and clinical characteristics were collected from medical records and using self-report questionnaires. Questionnaires were completed by participants prior to procedures. A research assistant was available to provide clarification about any of the items contained within the questionnaire.

### Measures

#### Capnography

The Respironics LoFlo sidestream CO_2_ sensor was used for capnography monitoring. The CO_2_ sampling cannula inserted into a sideport of an oxygen facemask with oxygen flow rates higher than 5 L/min or using nasal cannula with separate lines for sampling CO_2_ and delivering oxygen. The capnography waveform was displayed on the main physiological monitoring screen. A visible alert reading ‘No breaths detected’ would be triggered after 10 seconds of apnea but no other audible or visual alerts were set for capnography waveform abnormalities. As this study was observational, there were no restrictions or specific instructions provided to clinicians regarding how they should react to detection of any capnography waveform abnormalities. Nurses at the sites where data were collected are trained in advanced life support techniques and re-certified annually, where instruction on airway management is routinely provided. The researcher continuously observed the participant and capnography waveform and marked the onset and offset time of capnography waveform abnormalities in real-time. From these onset and offset times, a ‘respiratory state sequence’ for each patient was produced by classifying each second of the participant’s procedure into either a state of ‘normal breathing’, a state of apnea (no waveform or CO_2_ concentration of 0), a state of bradypneic hypoventilation (when the capnography-derived respiratory rate was less than 8) or a state of ‘hypopneic hypoventilation’ (when the capnography waveform amplitude was increased or decreased more than 10% from the baseline value). The classifications were selected based on a previous definition for capnography waveform abnormalities identified from the procedural sedation literature^11^. Waveform abnormalities corresponding to factors other than respiratory depression (i.e. talking or dislodged sampling cannula) were not included.

#### Transcutaneous carbon dioxide concentration

The researcher attached the Sentec VSign 2 sensor to the participant’s forehead using a multi-site attachment ring prior to sedation administration. The sensor was initially heated to a temperature of 45 °C then automatically dropped to 42 °C after stabilization. Once the Sentec Digital Monitoring System displayed a stabilized transcutaneous carbon dioxide (TcCO_2_) concentration, the monitor was covered with a drape so that it was not visible to research staff or clinicians during the procedure. The monitor was not used by the clinicians to guide treatment. Data was downloaded to a computer for analysis. Sentec TcCO_2_ monitoring provides continuous, accurate (mean bias 0.1 mmHg) and precise (95% limits of agreement within 9 mmHg) estimates of arterial CO_2_ (PaCO_2_)^12^. TcCO_2_ monitoring provides even more precise estimates of changes in PaCO_2_ (mean bias 0.03 mmHg, 95% limits of agreement −0.44 to 0.38 mmHg)^13^.

#### Oxygenation

Percentage of haemoglobin saturated with oxygen (SpO_2_) was measured continuously throughout procedures via the Sentec VSign 2 sensor and analyzed offline. SpO_2_ measurements recorded at at time that pulse rate was not detectable were filtered out because they were considered unreliable.

#### Recovery outcomes

We assessed for post-procedural cardiac (myocardial infarction or cardiac arrest [defined as ventricular fibrillation, asystole, electromechanical dissociation or ventricular tachycardia without cardiac output that required cardiopulmonary resuscitation and cardioversion]) and pulmonary complications (Postoperative PaO_2_ < 60 mmHg on room air, a ratio of PaO_2_ to inspired oxygen fraction <300, or arterial oxyhemoglobin saturation measured with pulse oximetry <90% and requiring oxygen therapy, treatment with antibiotics for a respiratory infection, pleural effusion confirmed by chest radiograph radiologist report, atelectasis confirmed by chest radiograph radiologist report, pneumothorax confirmed by chest radiograph radiologist report, bronchospasm defined as newly detected expiratory wheezing treated with bronchodilators) up to 30 days post-procedure from medical record review.

### Statistical analysis

Analyses were conducted with R, a language and environment for statistical computing, using TraMineR^14^. De-identified data and statistical code are available from a GitHub repository. Foundational to all subsequent analyses was the creation of a dissimilarity matrix by calculating pairwise distances between respiratory state sequences. A recent simulation study confirmed that there is no universally optimal approach to measure dissimilarities between categorical state sequences and, as such, the choice of a measure should be guided by the aspect of the state sequences that is most important to capture (i.e. duration, timing or sequencing)^7^. We considered that the duration of time spent in respiratory states throughout procedures was a more important feature than the timing or sequencing in which the respiratory states were observed. Dissimilarity measures based on calculating differences between state distributions over the whole length of sequences, such as the Euclidean and *χ*^2^ distance measures are the most sensitive to durations^7^. The Euclidean distance measure accounts for the overall proportion of time spent in states throughout the sequences. The *χ*^2^ distance measure weights the squared differences for each state by the inverse of the overall proportion of time spent in the states, which results in higher importance of rare states^7^. We calculated dissimilarity matrices for respiratory state sequences using the Euclidian and *χ*^2^ distances but ultimately chose the *χ*^2^ distance measurement as our primary approach because it was better able to distinguish differences in the duration of state of bradypnea, which occurred less frequently in our sample than the other states. We also applied the default normalization metric used for *χ*^2^ distance measures in the TraMineR package to account for differences in the length of respiratory state sequences arising from variability in procedural duration.

A hierarchical clustering algorithm was applied to the dissimilarity matrix in order to create a typology of common respiratory state sequences that were observed. The cluster package in R was used to identify clusters^15^. In particular, a Ward hierarchical clustering was performed using the dissimilarity matrix as input. A number of different potential solutions for cluster size (2 to 6) was tested and then measures of cluster quality and visualization tools, such as sequence index plots, were used to determine the final solution for the typology. A sequence index plot displays each participant’s respiratory state sequence with color-coded segments proportional to the length of time spent in a particular respiratory state were used to determine the final solution for the typology.

Multivariable distance matrix regression analysis (MDRMA) was used to examine associations between respiratory state sequence trajectories and explanatory variables. MDMRA measures the share of the dissimilarity between sequences that can be explained by a set of explanatory variables. The Pseudo-R^2^ is a measure of the strength of association, which can range from 0, indicating no association, to 1, indicating perfect association. It can be interpreted as the proportion of explained variance of all the factors in the model. The usual assumptions about distributions for parameters included in models that are typical of the classical significance tests are not applicable in this case. Instead, MDMRA uses a non-parametric approach with the statistical significance of the Pseudo-R^2^ estimated using permutation. We used 1000 permutations to build distributions to test the null hypotheses that there was no relationship between the distance matrix and the set of explanatory variables at the 5% significance level included in the MDMRA model (i.e. alpha of p < 0.05). The p-value indicates the chance that we would observe the Pseudo-R^2^ if there was no association between the distance matrix and the set of explanatory variables. We built a model to test associations between respiratory state sequences and a set of demographic and clinical variables including age, sex, medications for sedation (a Principal Components score for the total doses of midazolam and fentanyl was used because these two variables were highly correlated), diagnosis of obstructive sleep apnea, risk of obstructive sleep apnea (defined as low, moderate or high risk as per the recommended scoring from Cheung *et al*.^16–18^, diagnosis of COPD, admission status (elective same-day admission, emergency admission), body mass index, Charlson Comorbidity Index score and being a self-reported past or current smoker as well as baseline and peak TcCO_2_ concentrations, whether the participant received an intervention to support respiration, and hyperoxia (defined as the time in seconds during procedures with a SpO_2_ > 97%. We included hyperoxia in the model because prior studies have shown that higher PaO_2_ increases PaCO_2_ across a number of populations^19–21^. We tested the association between hypoxaemia (defined as number of seconds with SpO_2_ below 90%) and the respiratory state sequence distance matrix separately. This is because there were few instances of oxygen desaturation in the sample^22^.

## Results

From August 2016 to May 2018, 114 of the 129 (88%) patients who were screened prior to their procedure met the study eligibility criteria and consented to participate. Five participants (4.3%) were subsequently excluded because no sedation was administered during procedures. Respiratory state sequences were unable to be measured for four (4%) of the participants because the capnography equipment malfunctioned and for a further 3 (2.6%) participants because of researcher unavailability for intra-procedural data collection. The sample consisted mostly of patients undergoing permanent pacemaker implants or generator change procedures (n = 62; 61%) and was predominantly male (n = 67; 66%). The average age was 73 (SD 11). An overview of demographic and clinical characteristics for the whole sample is presented in Table 1. No severe cardiac or pulmonary complications occurred during follow up. One participant received antibiotic treatment for a respiratory infection within the follow-up period.

**Table 1:** Demographic and clinical characteristics by respiratory state sequence cluster type.

Summary Statistics	Total Sample (n = 102)	Normal breathing (n = 42)	Hypopnea (n = 38)	Apnea (n = 15)	Bradypnea (n = 7)
**Age**					
mean (sd)	72.95 ± 11.28	73.83 ± 11.63	73.21 ± 11.93	70.20 ± 10.46	72.14 ± 7.73
**Sex**					
Female	35 (34)	12 (29)	17 (45)	4 (27)	2 (29)
**Procedure**					
Permanent pacemaker implant or generator change	62 (61)	27 (64)	23 (61)	7 (47)	5 (71)
Implantable cardioverter defibrillator implant or generator change	10 (10)	4 (10)	4 (11)	1 (7)	1 (14)
Cardiac resynchronisation therapy	5 (5)	1 (2)	2 (5)	2 (13)	0 (0)
Atrial flutter ablation	8 (8)	3 (7)	3 (8)	2 (13)	0 (0)
Other arrhythmia ablation	13 (13)	5 (12)	4 (11)	3 (20)	1 (14)
Diagnostic electrophysiology study	3 (3)	2 (5)	1 (3)	0 (0)	0 (0)
Loop recorder implant	1 (1)	0 (0)	1 (3)	0 (0)	0 (0)
**Body Mass Index**					
mean (sd)	28.74 ± 5.36	29.92 ± 5.31	28.54 ± 5.09	25.77 ± 3.90	29.32 ± 8.29
**ASA classification status**					
One	11 (11)	4 (10)	5 (13)	2 (13)	0 (0)
Two	52 (51)	25 (60)	13 (34)	9 (60)	5 (71)
Three	32 (31)	9 (21)	18 (47)	3 (20)	2 (29)
Four	7 (7)	4 (10)	2 (5)	1 (7)	0 (0)
**Obstructive Sleep Apnea**					
Yes	25 (25)	10 (24)	7 (18)	4 (27)	4 (57)
No	77 (75)	32 (76)	31 (82)	11 (73)	3 (43)
**STOP-BANG Obstructive Sleep Apnea**					
**Risk Classification**					
Low	39 (41)	13 (32)	19 (53)	5 (38)	2 (29)
Moderate	26 (27)	12 (30)	9 (25)	3 (23)	2 (29)
High	31 (32)	15 (38)	8 (22)	5 (38)	3 (43)
**Chronic Obstructive Pulmonary Disease**					
Yes	16 (16)	9 (21)	4 (11)	1 (7)	2 (29)
No	86 (84)	33 (79)	34 (89)	14 (93)	5 (71)
**Past or present smoker**					
Yes	41 (41)	14 (33)	15 (41)	6 (40)	6 (86)
No	60 (59)	28 (67)	22 (59)	9 (60)	1 (14)
**Charlson Comoridity Index**					
mean (sd)	5.61 ± 2.40	5.52 ± 2.24	5.76 ± 2.09	4.60 ± 2.06	7.43 ± 4.39
**Midazolam total dose (mg)**					
max	6	6	6	5	3
mean (sd)	2.05 ± 1.10	1.88 ± 1.14	2.16 ± 1.10	2.20 ± 1.15	2.14 ± 0.69
**Fentanyl total dose (mg)**					
max	125	100	125	125	100
mean (sd)	55.40 ± 24.26	49.40 ± 22.42	58.58 ± 23.38	58.33 ± 29.38	67.86 ± 23.78
**TcCO**_**2**_ **at baseline (mm Hg)**					
mean (sd)	37.48 ± 4.61	37.09 ± 4.12	38.43 ± 4.64	36.27 ± 5.43	37.26 ± 5.38
**TcCO**_**2**_ **peak (mm Hg)**					
max	70.5	55.8	70.5	62.9	57.8
mean (sd)	47.23 ± 6.68	44.58 ± 4.95	49.54 ± 7.06	47.73 ± 7.55	49.56 ± 7.27

Legend: ASA = American Society of Anesthesiology; STOPBANG = The snoring, tiredness, observed apnea, high blood pressure, Body mass Index, age, neck circumference, and male gender (STOP-Bang) questionnaire; mg = milligram; mcg = microgram; TcCO_2_ = transcutaneous carbon dioxide concentration; mmHg = pressure measured in millimetre of mercury; sd = standard deviation.

### Interventions used to support respiration

All participants received prophylactic supplemental oxygen prior to sedation administration with either a face mask or nasal prong delivery system. Most participants were hyperoxic (defined as SpO_2_ > 97%) for the majority of procedures (median 93% of procedure time in hyperoxic state). Only three (3%) patients experienced oxygen desaturation below 90% for longer than 30 seconds. The longest period of desaturation below 90% was for 80 seconds. A total of 49 interventions were applied to support respiration to thirteen participants (13%). In no cases was sedation reversal or placement of airway adjuncts used. Interventions were mostly applied during a state of apnea (n = 35, 71%). Verbal breathing cues were the most common intervention applied (n = 41, 84%), with other interventions being airway realignment or jaw support applied three times and an increase in oxygen supplementation used for 5 (5%) participants.

### Identification of typical respiratory state sequences from cluster analysis

**Figure 1:**
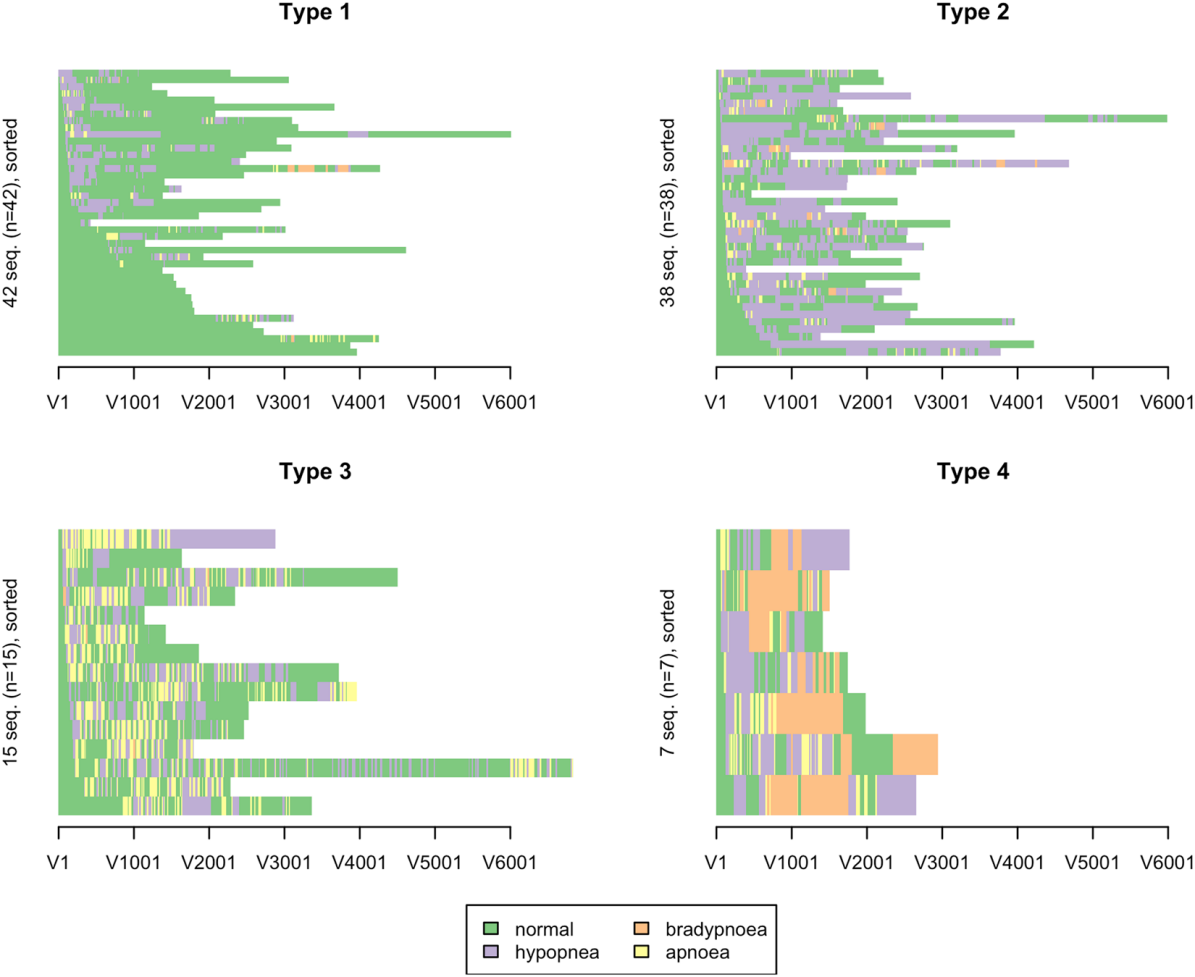
Sequence index plot for each cluster type. Clusters were characterized by the dominant respiratory state. Type 1 was classified as the’normal breathing’ pattern, Type 2 as the’hypopneic hypoventilation’ pattern, Type 3 as the’apneic’ breathing pattern and Type 4 as the’bradypneic hypoventilation’ pattern.

Figure 1 displays sequence index plots for each of the four identified clusters. The demographic and clinical characteristics of the participants grouped by cluster are presented in Table 1. One major cluster consisting of 41% of the sample (n = 42) was characterized by a predominance of the state assigned ‘normal breathing’ (i.e. no capnography waveform abnormalities). The next most common cluster type was characterized by capnography waveform abnormalities indicative of hypopneic hypoventilation for the majority of procedures (n = 38; 37%). Smaller proportions of the sample were assigned to the remaining clusters. Fifteen participants (15%) who experienced frequent and at times prolonged apneic periods comprised one of the clusters. The smallest cluster (n = 7; 7%) was characterized by the state of bradypneic hypoventilation.

### Inter-individual differences in respiratory state sequences from multivariable distance matrix regression analyses

**Figure 2:**
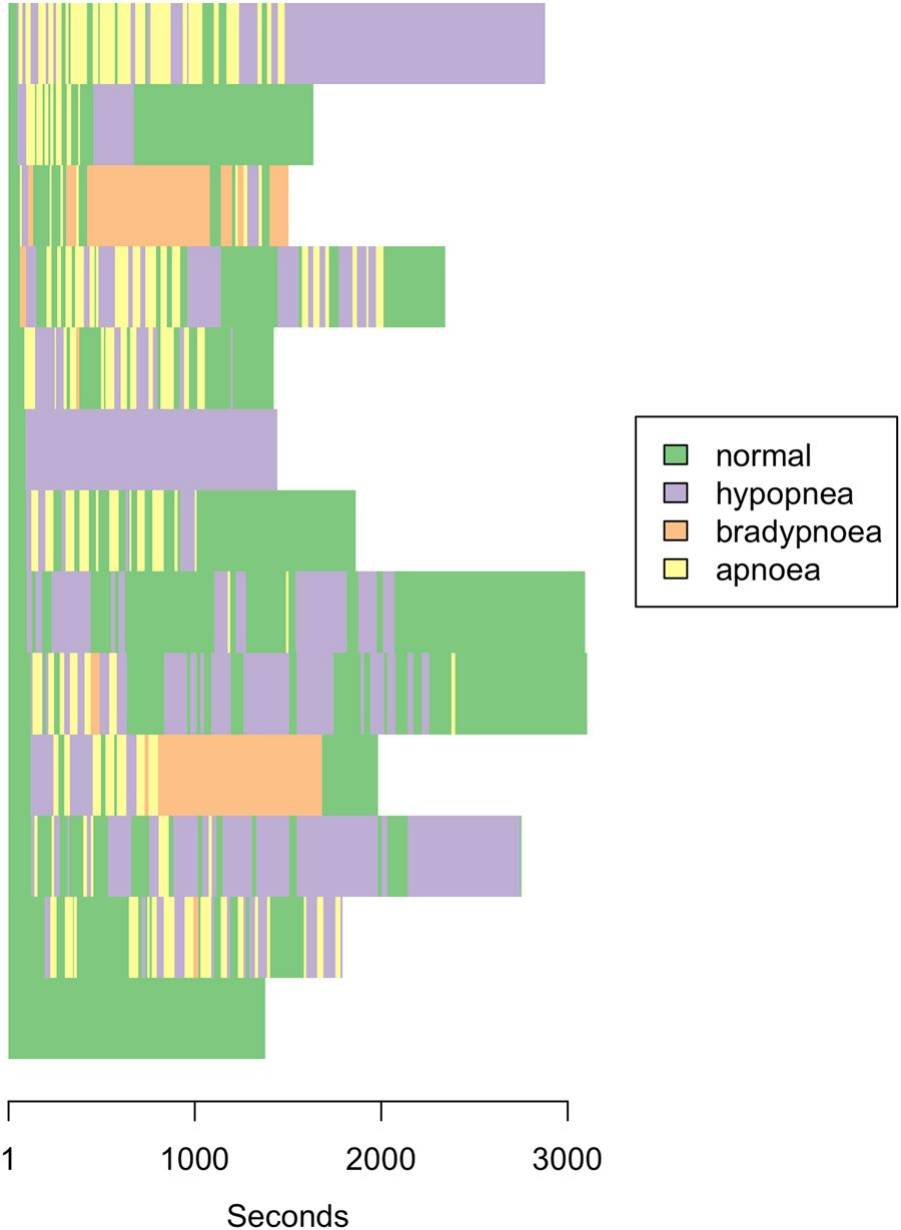
Sequence index plot of patients who received an intervention to support breathing (n = 13).

The MDMRA model was built to examine the strength of associations between inter-individual differences in respiratory state sequences with demographic and clinical characteristics (Table 2). The global statistics for the model indicate that the set of variables provided significant information about the dissimilarities between respiratory state sequences. The variables explained 28.2% of the variation in inter-individual differences in respiratory state sequences. Significant covariates included having a diagnosis of OSA, being a past or present smoker and the Charlson Comorbidity Index score. However, each of these covariates explained only modest amounts of variation. Peak TcCO_2_ concentrations explained 4.1% of the variation in the dissimilarity matrix, equating to 15% of the total variance explained by all the variables included in the model. Receiving an intervention (verbal breathing cues, increased oxygen, airway realignment) was significantly associated with respiratory state sequence dissimilarities. This variable explained 5.6% of the variation in respiratory state sequences (p = 0.001). The large inter-individual variability in respiratory state sequences among patients who received intervention is clear from the sequence index plot presented in Fig. 2. Oxygenation status was not associated with respiratory state sequence trajectories.

**Table 2:** Multivariate distance matrix regression analysis

Variable	Pseudo F	Pseudo R^2^	Proportion explained	p value
Peak TcCO_2_	4.358	0.043	0.152	0.002
Baseline TcCO_2_	1.525	0.015	0.053	0.140
Obstructive Sleep Apnea	2.337	0.023	0.082	0.023
Age	0.689	0.007	0.024	0.674
Body mass index	0.936	0.009	0.033	0.460
Sex	0.599	0.006	0.021	0.800
Day surgery admission	1.270	0.012	0.044	0.243
PCA factor 1 of total sedation doses	1.404	0.014	0.049	0.185
PCA factor 2 of total sedation doses	1.608	0.016	0.056	0.134
Past or present smoker	2.793	0.027	0.097	0.010
Chronic Obstructive Pulmonary Disease	1.136	0.011	0.040	0.284
Charlson comorbidity index score	2.626	0.026	0.092	0.021
Patients who received intervention to support respiration	5.700	0.056	0.199	0.001
STOPBANG (low, intermediate or high risk)	0.896	0.018	0.063	0.541
Emergency admission	0.800	0.008	0.028	0.576
Time above SpO_2_ 97%	0.477	0.005	0.017	0.898
Total	1.686	0.282	1.000	0.001

Legend: PCA = Principal components analysis; STOPBANG = The snoring, tiredness, observed apnea, high blood pressure, Body mass Index, age, neck circumference, and male gender (STOP-Bang) questionnaire; SpO_2_ = Percentage of hemoglobin saturated with oxygen; TcCO_2_ = transcutaneous carbon dioxide concentration.

## Discussion

This study used a novel approach to examine capnography waveform abnormalities observed during nurse-administered procedural sedation and analgesia. Descriptive analysis of the respiratory state sequences clearly demonstrated that capnography waveform abnormalities are frequent and prolonged in this typical (mostly elder) population of patients undergoing cardiac procedures who received sedation with small bolus doses of midazolam and fentanyl. Applying a clustering algorithm to group similar respiratory state sequences revealed insights into common patterns of ventilatory responses to the medication used for procedural sedation. That a considerable proportion of the sample (about 15%) spent long durations of the procedures experiencing apnea is an important finding. Further research into predicting ahead of time if a patient is likely to fall into this particular respiratory state sequence cluster type could be useful information for tailoring treatment during sedation. For example, adding ketamine to the combination of medications used for procedural sedation was found to significantly improve ventilation status^23^. There are also experimental agents that show promise in averting opioid-induced respiratory depression that may in the near future translate into clinical practice after further investigation of their efficacy and toxicity^24^. High flow nasal oxygen is also increasingly regarded as a potentially useful new technology for patients at risk of inadequate oxygenation and ventilation status during sedation^25^.

It should be noted that due to the descriptive nature of cluster analysis it is not possible to assume that there was an underlying ‘model’ generating the types of respiratory state sequences that characterized these clusters^22^. However, the results from the multivariable distance matrix analyses that we undertook suggest that there are factors that provide unique, yet modest, contributions to differences in respiratory state sequence trajectories. These variables included the Charlson Comorbidity Index, having a diagnosis of obstructive sleep apnea or being a current or past smoker.

A noteworthy result from this study was that total amounts of sedation were not associated with inter-individual differences in trajectories of post-sedation respiratory states. It is known that there is wide variability in the amount of sedative and analgesic medications required to induce moderate sedation^26^. Also, our previous investigations in this population focusing on oxygen saturation also did not identify associations between respiratory depression and total doses of sedation^27^. In a study of predictors of apnea in patients receiving procedural sedation in the emergency department, age but not sedation dose was significantly associated with apnoea^28^.

Some useful insights were gained from this study into how nurses use capnography in their practice. Previous randomized controlled trials that compared the use of capnography with usual practice had varied protocols for the capnography waveform abnormalities that triggered intervention^4^. Most involved an alarm or alert being triggered after a capnography waveform abnormality indicative of bradypnea, hypoventilation or apnea was observed for a pre-specified period of time. For example, a recent randomized trial that investigated the utility of capnography for monitoring of moderate sedation during endoscopy and colonoscopy involved alarm triggers when the respiratory rate was less than 8 per minute, apnea was detected for more than 5 seconds or if there was a >75% reduction from the baseline value for >10 seconds^29^. In contrast to the high frequency in which capnography waveform abnormalities such as these were observed in our study, few patients received interventions to support their ventilation. Furthermore, interventions were minor, comprising mostly of verbal breathing cues. Yet, oxygenation status was not compromised, with no serious periods of desaturation observed. In our study, nearly all patients received oxygen supplementation through a face mask at 6 liters per minute. A large randomized controlled trial of capnography for sedation that administered supplemental oxygen at high flow rates through a face mask from the outset of procedures identified no difference in the incidence of oxygen desaturation events between the capnography (7 of 501 participants; 1.4%) and no capnography (9 of 485 participants; 1.9%) arms (p = 0.569)^30^. The results of this trial may explain why the participants in our study who were observed to have capnography waveform abnormalities but did not receive any interventions to support ventilation were able to maintain oxygen saturations within normal limits.

From multivariable distance matrix regression analyses, we identified that respiratory state sequences of patients who received a clinical intervention to support respiration were more similar to each other than the respiratory state sequences of participants who did not receive an intervention. However, the proportion of variation in respiratory state sequence dissimilarity that this variable explained was small (5.7%). This finding is consistent with recent prior research conducted in sedated patients from a different context. Krauss and colleagues^28^ investigated associations between capnography waveform abnormalities observed during emergency department procedural sedation in adults with propofol or ketamine and a clinician’s propensity to initiate interventions to support ventilation. There were no distinguishing features that separated occurrences of apneic periods that did and did not trigger a clinical intervention^28^. Therefore, our study provides further evidence that the decision to intervene in response to detection of capnography waveform abnormalities in the clinical practice is complex and varies considerably between clinicians.

Specific areas that require further investigation were identified from this study. We found that TcCO_2_ concentrations were associated with respiratory state sequence trajectories. However, the clinical significance of periods of hypercapnia during procedural sedation with concomitant normal oxygenation is unclear. Further research should be conducted to determine if accurate predictions can be made from respiratory state sequences to potentially allow for earlier detection and initiation of treatment to attenuate the effects of sedation on sedation-induced CO_2_ retention. Furthermore, cluster analysis identified a small group of patients who experienced long periods of bradypneic hypoventilation. Further larger studies would be required to determine a more precise estimate of how common this particular pattern of response to sedative medications is and also to explore the clinical significance of long periods of reduced respiratory rates during procedural sedation.

A limitation of this study to note is the potential for selection bias, arising from the non-randomized design. Also, data were collected at only two sites. Clinicians at other institutions may have different approaches for how they use capnography to detect and treat respiratory depression during nurse-administered procedural sedation. Participants in this study received small, incremental bolus doses of midazolam and fentanyl for sedation. Results should not be generalized outside of this context and further research with a larger sample size is required to increase confidence in the results. There were no serious desaturation episodes and no severe post-procedure cardiac or pulmonary adverse events in our study population and so we were unable to explore whether these events are more prevalent with particular respiratory state sequence trajectories. Replication of this study in a cohort with a higher prevalence of clinically significant desaturation, for example patients undergoing bronchoscopy, or recruiting a larger dataset would permit the relationship between respiratory state sequences and clinically important deterioration to be explored in more detail.

We were unable to include a small number of recruited participants in this analysis due to equipment malfunction and researcher unavailability for data collection. Although it has been used in prior capnography research^31^, we decided that automatic downloading of capnography waveforms from stored monitor data would be unreliable. Without direct observation it would not have been possible to identify which measurements were inaccurate due to factors such as a dislodged CO_2_ sampling cannula. As the population under study were receiving moderate sedation, it is also possible that capnography waveform abnormalities may be observed when patients were intentionally holding their breath (when instructed by the proceduralist for imaging reasons) or when talking. For that reason, we categorized the ‘respiratory states’ in real time by the same observer. The potential for risk of bias arising from the fact that the observer was an author of the study should be considered. Potentially, the author may have been expecting a certain result and thus be more likely to dismiss data supporting the opposite result in some cases. It should be noted however that the direct observation approach mimics how clinicians monitor ventilation in practice. Therefore, the respiratory state sequences should be viewed as accurate representations of the patterns of ventilation in patients receiving sedation, which are detectable by an observer classifying capnography waveform abnormalities. We used criteria for classifying waveform abnormalities that have been used in previous research. It is uncertain if different results may have been observed from the MDMRA and clustering algorithms to develop typologies of similar respiratory state sequences if other classification criteria were used (eg different ETCO_2_ cut-offs for classifying hypoventilation or different respiratory rate cut-off for classifying bradypnea).

A further limitation to be noted is that a formal sample size calculation for undertaking MDMRA was not performed before commencement of this study, as it was originally planned to perform latent class analysis. For this reason we can not exclude the possibility of type II error regarding the null hypothesis that was tested in the MDMRA (i.e. that were was no association between the predictor variables and the paired distances in respiratory state sequences). Post-hoc power analysis are discouraged so we did not attempt any formal evaluations to determine if the sample size was sufficient for MDMRA^32^. A simulation study has identified evidence that the accuracy of permutation methods used to test hypotheses in MDMRA are acceptable in sample sizes similar to ours (n = 100)^9^.

## Data Availability

De-identified data and statistical code are available from a GitHub repository.
